# Phenotypic selection on floral traits in the arctic plant *Parrya nudicaulis* (Brassicaceae)

**DOI:** 10.1002/ece3.8624

**Published:** 2022-03-01

**Authors:** Matthew L. Carlson, Justin R. Fulkerson

**Affiliations:** ^1^ 3291 Alaska Center for Conservation Science University of Alaska Anchorage Anchorage Alaska USA; ^2^ 3291 Biological Sciences Department University of Alaska Anchorage Anchorage Alaska USA

**Keywords:** Alaska, floral evolution, flower color, flower number, phenotypic selection, pollen limitation, seed production

## Abstract

The evolution of floral traits is often attributed to pollinator‐mediated selection; however, the importance of pollinators as selective agents in arctic environments is poorly resolved. In arctic and subarctic regions that are thought to be pollen limited, selection is expected to either favor floral traits that increase pollinator attraction or promote reproductive assurance through selfing. We quantified phenotypic selection on floral traits in two arctic and two subarctic populations of the self‐compatible, but largely pollinator‐dependent, *Parrya nudicaulis*. Additionally, we measured selection in plants in both open pollination and pollen augmentation treatments to estimate selection imposed by pollinators in one population. Seed production was found to be limited by pollen availability and strong directional selection on flower number was observed. We did not detect consistently greater magnitudes of selection on floral traits in the arctic relative to the subarctic populations. Directional selection for more pigmented flowers in one arctic population was observed, however. In some populations, selection on flower color was found to interact with other traits. We did not detect consistently stronger selection gradients across all traits for plants exposed to pollinator selection relative to those in the pollen augmentation treatment; however, directional selection tended to be higher for some floral traits in open‐pollinated plants.

## Graphical abstract

We quantified phenotypic selection on floral traits in two arctic and two subarctic populations of *Parrya nudicaulis*, and measured selection in plants in both open pollination and pollen augmentation treatments to estimate selection imposed by pollinators. Seed production was pollen limited; however, strength of selection on floral traits exposed to pollinator‐mediated selection was not consistently greater, and selection in the Arctic populations was not consistently greater.

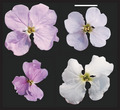

## 1 INTRODUCTION

Selection for improving the pollinator‐mediated export and receipt of pollen to flowering plants is recognized to have been the primary driver in the vast and rapid diversification of floral form (Ashman & Morgan, 2004; Caruso et al., 2019; Fægri & van der Pijl, 1979; Fenster et al., 2004, 2015; Grant & Grant, 1965; Harder & Johnson, 2009; Phillips et al., 2020; Schiestl & Johnson, 2013). Pollinators have been shown to discriminate among differences in floral traits such as inflorescence size, flower size, flower orientation, floral scent, and nectar production (Campbell et al., 2012, 2016; Fenster et al., 2004; Gervasi & Schiestl, 2017; Hodges et al., 2002; Parachnowitsch & Kessler, 2010; Sandring & Ågren, 2009; Schiestl et al., 2011). Flower color is also important for pollinator attraction, and pollinator foraging intensity is commonly associated with variation in flower pigmentation (Brunet et al., 2021; Hodges et al., 2002; Irwin & Strauss, 2005; Jones & Reithel, 2001; Medel et al., 2003; Schemske & Bradshaw, 1999; Streisfeld & Kohn, 2007; Trunschke et al., 2021). The fitness consequences due to differential attraction to pollinators based on phenotypic variation can be severe (cf. Alexandersson & Johnson, 2002; Caruso et al., 2019; Parachnowitsch et al., 2012; Schemske & Bradshaw, 1999; Stanton & Preston, 1988). Direct estimates of selection by pollinators on flower color and other floral traits are not common, despite the assumption that current patterns in adaptive evolution are in fact reflections of pollinator‐mediated selection (Campbell & Bischoff, 2013; Campbell et al., 2012; Parachnowitsch & Kessler, 2010; Sandring & Ågren, 2009; see reviews in Caruso et al., 2019 and Trunschke et al., 2021).

Selection on floral traits is expected to be greatest when plants are limited by pollen availability (Bartkowska & Johnston, 2012; Benkman, 2013; Caruso et al., 2019; Haig & Westoby, 1988; Sletvold & Ågren, 2010; Sletvold et al., 2017; Trunschke et al., 2017). Pollen limitation is predicted to be strongest in habitats with low and stochastic pollinator availability (Ashman et al., 2004; Burd et al., 2009; Garcia‐Camacho & Totland, 2009). The severe climate of wet, windy, and cooler temperatures limits the flying time and flower visitation rates of pollinators of arctic and alpine tundra habitats and, to a lesser extent, subarctic habitats (Arroyo et al., 1985; Bergman et al., 1996; Hocking, 1968; Kevan et al., 1993; Totland, 1994). Moreover, tundra and taiga biomes have a low abundance and diversity of pollinators; this is particularly striking in the Arctic (Arroyo et al., 1985; Bergman et al., 1996; Elberling & Olesen, 1999; Kevan et al., 1993; Totland, 1994). Strong pollen limitation can have important evolutionary consequences where species that have, or evolve, mechanisms for reproductive assurance (i.e., increasing ability to self‐fertilize independently of any pollen vector) are expected to have higher fitness (Ashman et al., 2004; Harder & Aizen, 2010; Morgan & Wilson, 2005; Porcher & Lande, 2005). Selection is therefore expected to favor traits that either increase selfing (e.g., reduced anther–stigma separation) or enhance pollinator attractiveness to increase pollen receipt (e.g., increased flower size and nectar secretion rates) (Ashman & Morgan, 2004; Campbell & Bischoff, 2013; Harder & Aizen, 2010; Johnston, 1991; Totland, 2001).

While arctic plant–pollinator ecological relationships are studied to some extent (see Carlson et al., 2008; Cirtwill et al., 2018; Kevan, 1972; Koch et al., 2020; Lundgren & Olesen, 2005; Molau, 1993; Robinson et al., 2018; Tiusanen et al., 2016; Urbanowicz et al., 2018), the selective pressures and evolutionary processes have been largely overlooked. The lack of attention is possibly attributed to the arctic (and alpine) angiosperm flora typically being considered depauperate in terms of investment in animal pollination; the flora being composed of wind‐pollinated, apomictic, and self‐fertilizing plants, with pollinators often presumed to be of trivial importance to plant reproduction (Billings, 1974; Billings & Mooney, 1968; Bliss, 1962; Johnson, 1969; Lloyd, 1980; Löve, 1959; Mosquin, 1966; Richards, 1997). Flowers of arctic species have even been suggested to be vestigial organs; remnants of the evolutionary past, inherited from ancestors to the south (Löve, 1959; Mosquin, 1966). Contrary to these assertions, pollinators have been shown to be necessary for seed production in numerous arctic and subarctic alpine plant species, and many tundra plants have mixed mating systems with intermediate levels of outcrossing (see review in Goodwillie et al., 2005; Koch et al., 2020; Urbanowicz et al., 2018) and with some arctic taxa possessing self‐incompatible systems (Bingham, 1999; Fulkerson et al., 2012; Grundt et al., 2005; Kevan, 1972; Tikhmenev, 1985). Furthermore, the non‐graminoid arctic vascular flora as a whole contains a relatively high percentage of anthocyanin‐pigmented taxa with many capable of producing nectar and scent (Jaakola & Hohtola, 2010; Whittall & Carlson, 2009), which is suggestive of pollinator‐mediated selection contributing to the maintenance of those traits.

**FIGURE 1:**
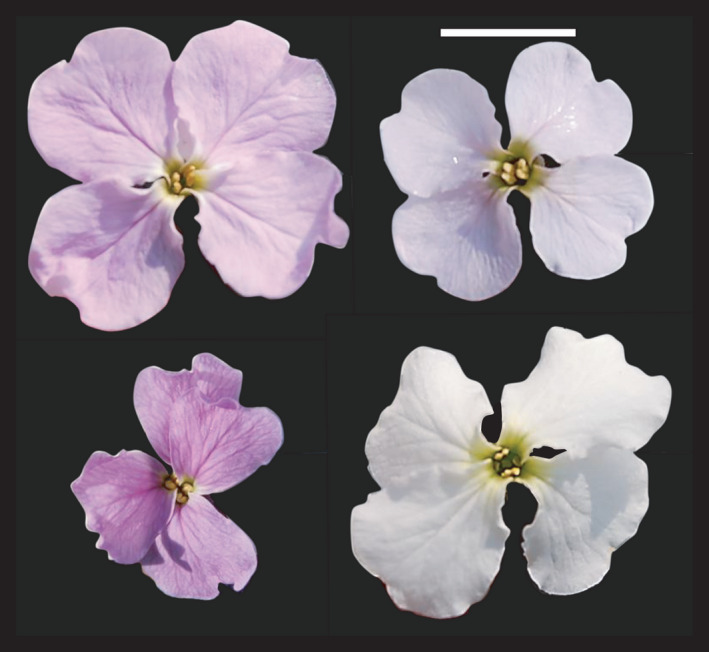
*Parrya nudicaulis* flowers from Eagle Summit, showing the broad range of floral pigmentation and corolla size. Scale bar = 1 cm

Here, we estimate the magnitude of phenotypic selection on floral traits (flower number, petal size, corolla depth, anther height, and flower color), using seed set as a proxy for fitness in the arctic and subarctic mustard, *Parrya nudicaulis* (Brassicaceae). High within‐population variation in flower size, petal orientation, and pigmentation is common in *P*. *nudicaulis* (Figure 1). This species is largely pollinator dependent and severely pollen limited (Fulkerson et al., 2012). With the use of pollen augmentation and control treatments in *P*. *nudicaulis* (see Sandring & Ågren, 2009), we predict that the strength of pollinator‐mediated selection is greater than non‐pollinator‐mediated selection on floral traits associated with increased pollinator attraction. Last, as pollinator service is expected to be of poorer quality in arctic relative to subarctic populations, we predict that phenotypic selection on floral traits is greater in the more northerly populations.

## 2 MATERIALS AND METHODS

### 2.1 Study system

*Parrya nudicaulis* L. Regel (Brassicaceae) is found from northeastern Asia, across Alaska and to the western Canadian Arctic Archipelago (Al‐Shehbaz, 2010; Hultén, 1968). Flowering occurs in late May to mid‐June in subarctic sites in Alaska and several weeks later on the Arctic Coastal Plain. At reproductive maturity, this perennial herb produces a single raceme of 8–14 flowers, which normally persists between 10–14 days with individual flowers senescing after 3 days. Flowers are protandrous; the upper anthers dehisce shortly after the flowers open, followed by the lower two anthers within approximately 12 h, and the stigma becomes bilobed and receptive during the second day. Flower color of *P*. *nudicaulis* is highly variable among individuals in many populations (Butler et al., 2014). While the hue is quite consistent, the lightness values range dramatically among individuals. Flowers range from pure white, and produce no anthocyanins, to dark violet with substantial anthocyanin production (Dick et al., 2011). Most flowers emit a sweet fragrance comparable to *Syringa* species. Nectar is secreted at the base of the corolla and less than 4 µl is produced in plants bagged for 24 h (Fulkerson et al., 2012). Floral visits to *P*. *nudicaulis* at the studied populations in Alaska are infrequent (mean of 0.14 and 0.58 visits/flower/hour in 2009 and 2010), and although a diversity of visitors drink nectar and collect pollen on *P*. *nudicaulis*, muscid and syrphid flies make up the largest proportion of floral visitors (Fulkerson et al., 2012).

This study was conducted at two arctic and two subarctic populations. The Galbraith site (68°27′N, 149°33′W, 880 m elevation) and Ivishak site (69°20′N, 148°45′W, 280 m elevation) are located on the foothills of the Arctic Coastal Plain. Both the Galbraith and Ivishak sites are found within the Northern Alaska Arctic Floristic Provence and are a graminoid tundra habitat dominated by tussock sedge, dwarf shrubs, and moss and lichens (Raynolds et al., 2005). The two subarctic sites were Eagle Summit (65°28′N, 145°25′W, 1,100 m elevation) and Twelve‐Mile (65°24′N, 145°44′W, 680 m elevation) and are located in the White Mountains of interior Alaska and consist of mesic forb‐ericaceous shrub tundra above treeline.

We stratified sampling of individuals by three broad color categories visible to the human eye: white, light violet, and dark violet. The Eagle Summit and Twelve‐Mile populations in the subarctic consisted of relatively equal proportions of individuals in each color category. The two arctic populations contained relatively few pure white individuals, thus all white individuals in these populations were sampled.

Phenotypic selection was estimated on a total of 41 individuals at Galbraith, 64 individuals at Ivishak, and 42 individuals at Twelve‐Mile in 2009. In 2010, 57 individuals were sampled at Ivishak. We contrasted pollinator‐mediated selection with non‐pollinator selection at a single subarctic population (Eagle Summit) in 2010. At this site, plants were randomly assigned to open‐ (129 individuals) or pollen augmentation treatments (83 individuals), which is designed to remove the component of phenotypic selection imposed by pollinators (for more discussion of this approach, see Sandring & Ågren, 2009). Treatments and measurements of Eagle Summit occurred at the beginning of *P*. *nudicaulis* flowering season in early June. Infructescences were collected at the end of July, prior to seed dehiscence.

### 2.2 Pollination treatments

To remove the component of phenotypic selection due to pollinator visitation, mixed pollen from at least 10 haphazardly selected individuals that were >10 m distance from the recipient were used to hand‐pollinate flowers. Phenotypic selection was not estimated from plants that served as pollen donors. Manipulated flowers were marked with a small amount of “puffy paint” at the base of the pedicel. Every flower was hand‐pollinated every day, until there were signs of flower senescence to ensure that stigma receptivity was not missed. Supplemental pollen added to the entire inflorescence reduces the chance of differential resource allocation interfering with the detection of pollen limitation (Ashman et al., 2004; Knight et al., 2006; Zimmerman & Pyke, 1988). The fate of all flowers was followed to estimate probability of seed set and fecundity for each plant.

### 2.3 Phenotypic measurements

We used the measurements of six floral traits that we expected could be under pollen‐mediated selection: flower number, petal width, petal length, corolla depth, anther height, and flower color. Petal length was highly correlated with petal width (*r* = 0.65 *p* < .001), and both measurements were reflective of flower size, and therefore to reduce multicollinearity, petal length was not included in the analysis. Pistil height was correlated with corolla depth and the stigma became receptive when it neared the corolla opening; we did not measure pistil position to avoid contact or damage to the stigma. We counted the total number of flowers produced at the end of the flowering season. All other traits were measured at anthesis when the flowers were fully open and anthers were accessible to pollinators. We measured the width and lengths of the largest petal, corolla depth, and height of the tallest anthers to the nearest 0.01 mm with a digital caliper at Eagle Summit, Galbraith, and the 2009 Ivishak plants. To capture a large enough sample with limited time, at the Twelve‐Mile and 2010 Ivishak populations, we measured corolla depth and anther height with digital calipers, but we measured petal length and width using digital photographs of individual flowers with a scale bar; measurements were subsequently made in ImageJ (Rasband, 1997‐2018) image analysis software. Means and variance measurements of all traits are summarized in Tables S1‐S5.

A Royal Horticultural Society Colour Chart (RHS, 2007) was used to quantify the variation in flower color between plants at the time of anthesis. Using this chart, however, limits the factor of “color” to categorical data. To determine lightness values of the color chips, we used the techniques followed by Fulkerson et al. (2012) to create CIE L* values: L* values range from 0 to 100, where “0” is black or “near‐black” and “100” is white or “near‐white” (see Stevens et al., 2007; Voss, 1992). Flower color was characterized by a total of 18 color chips in these populations and ranged from L* value 59.5 to 99.5. *Parrya nudicaulis* petals fall within a narrow range of purple–violet of the RHS Colour Chart, and L* is highly correlated with anthocyanin concentration (J. B. Whittall, unpublished data).

### 2.4 Selection analysis

The strength and direction of selection on the floral traits were measured using a multivariate regression analytic framework (Lande & Arnold, 1983). We used variance‐standardized partial linear regression coefficients to estimate the strength of directional selection on traits independent of all other measured traits (i.e., selection gradients, *β_σ_*) (Lande & Arnold, 1983). Additionally, we calculated mean‐standardized selection coefficients (*β_μ_*), as this metric has been shown to avoid the problem of conflating selection and variation and it is particularly useful for summarizing the strength of selection for diverse traits, and for facilitating a more accurate estimate of response to selection (see Hereford et al., 2004). Trait standardizations were made for the individuals used in each regression model. Mean‐standardized results are presented in the Tables S1–S5. The number of individuals was not sufficient to measure nonlinear selection (convex or concave) for all populations, although the sample size approached recommended levels for Eagle Summit open‐pollinated and pollen augmentation treatments (see Walsh & Lynch, 2018). We therefore quantified nonlinear selection and correlational selection for variance‐standardized traits at Eagle Summit between pairs of traits using quadratic (*γ_ii_*) and 15 cross‐product (*γ_ij_*) terms in the regression model (Sandring & Ågren, 2009). These regression coefficients were multiplied by 2 to derive the nonlinear selection coefficients (Stinchcombe et al., 2008). Fitness was estimated by two separate values: the probability of producing seed and fecundity for those individuals which produced seed. Thus, the first fitness metric separates plants that had reproductive failure to those that reproduced (i.e., either received insect visitation or self‐fertilized). The second fitness metric encompasses the quality and quantity of pollination of plants that did reproduce. This approach also facilitates use of different regression models without violating assumptions. These fitness values were relativized by dividing by the population mean. Multiple logistic regression was used to estimate selection on the probability of seed set due to the dichotomous nature of this fitness measure (Janzen & Stern, 1998). Binomial logistic regression coefficients were transformed into linear regression coefficients using the methods of Janzen and Stern (1998). Secondarily, we measured selection gradients on those individuals that did set seed at the experimental population at Eagle Summit and Ivishak using standard multiple regression methods. Contrasts in the magnitude and direction of selection gradients between open‐pollinated and pollen‐augmented treatments and among arctic and subarctic populations were compared with means and 95% confidence intervals to avoid the pitfalls of null hypothesis significance testing (Anderson et al., 2000; Fidler et al., 2006; Rinella & James, 2010). All analyses were conducted using R version 2.12 (R Development Core Team, 2011).

## 3 RESULTS

Pollen limitation was evidenced by a nearly fourfold increase in seed production in pollen‐augmented plants at Eagle Summit compared to open‐pollinated plants (10.96 ± 1.19 in pollen‐augmented plants relative to 2.47 ± 0.51 SE seeds/plant in open‐pollinated plants, respectively). The pollen limitation index (see Lavi & Sapir, 2015; Trunschke et al., 2017) was 0.75. The other subarctic population, Twelve‐Mile, produced 5.33 ± 0.91 SE seeds/plant. In 2009, the arctic sites at Galbraith and Ivishak produced 2.00 ± 0.59 SE seeds/plant and 6.26 ± 0.87 SE seeds/plant, respectively. In 2010, seed production was 9.95 ± 0.40 SE seeds/plant at the Ivishak population.

### 3.1 Phenotypic selection on floral characters

**FIGURE 2:**
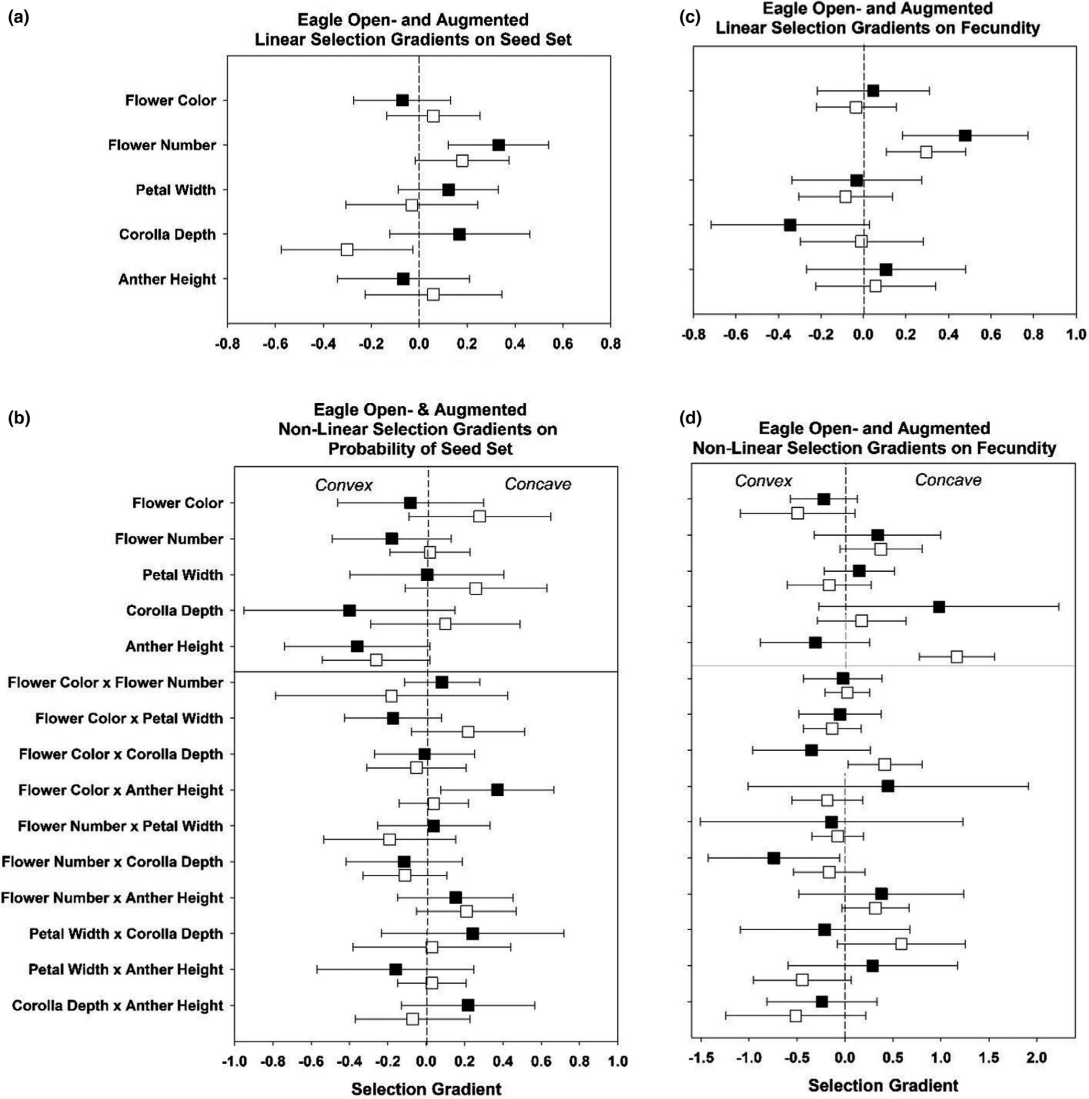
Variance‐standardized linear (a and c) selection gradients (*β_σ_*) and non‐linear (b and d) selection gradients (*γ_σ_*) for plants subjected to pollen‐mediated selection (black squares) and pollen‐augmented plants (open squares) on probability of seed set and fecundity at Eagle Summit 2010. Bars display the 95% CI

**FIGURE 3:**
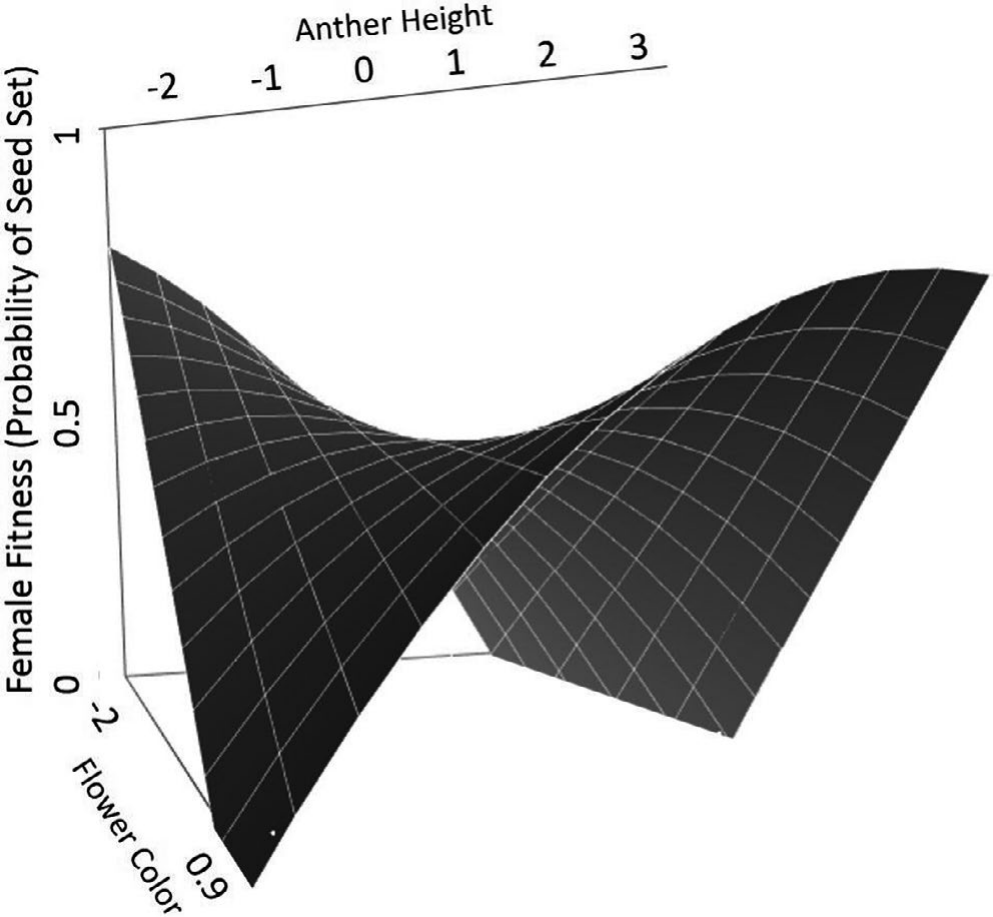
Relationship of relative fitness (probability of seed set) to flower color and anther position in pollinator‐mediated selection treatment at Eagle Summit. Trait axes are in units of standard deviations. Darkly pigmented flowers are represented by negative values of greater magnitude, and lighter pigmented and unpigmented flowers have positive values of greater magnitude. Positive anther positions indicate a higher and generally more exserted anther position relative to the base of the corolla tube; negative anther positions indicate plants with lower than average anther position. The probability of seed set was highest for dark pigmented flowers with short anther position and light flowers with exserted anthers

**FIGURE 4:**
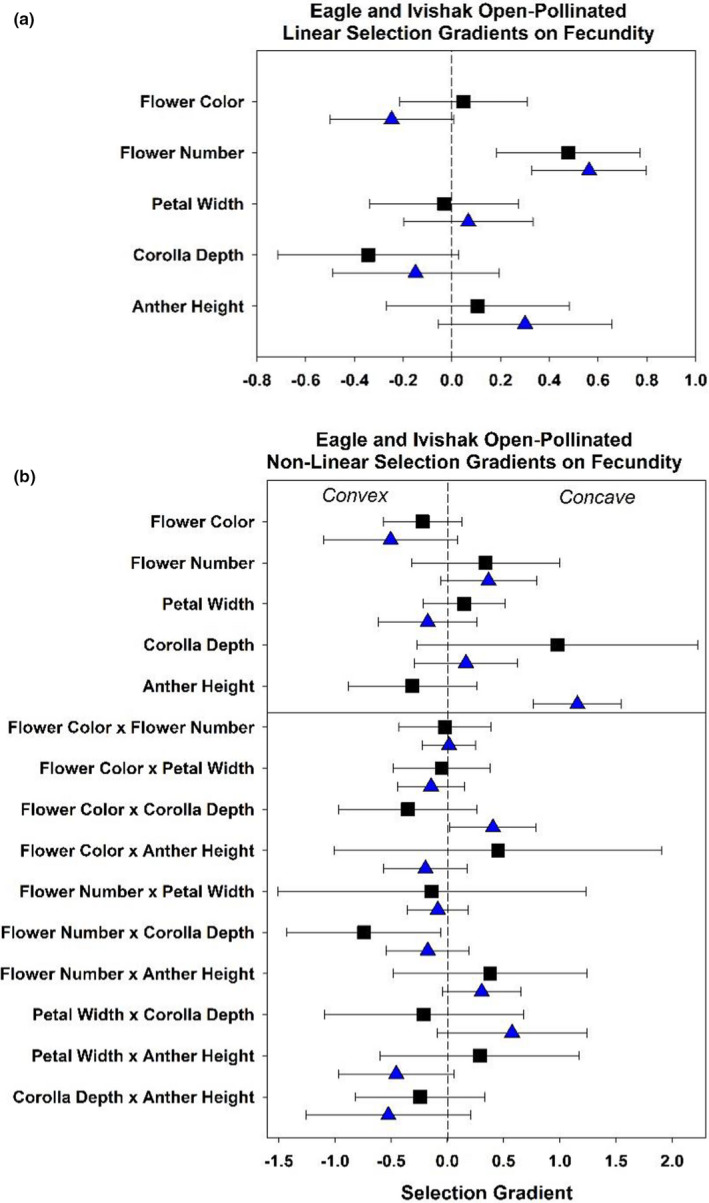
Variance‐standardized linear (*β_σ_*) (above) and nonlinear (*γ_σ_*) (below) selection gradients on fecundity for subarctic Eagle Summit 2010 (black squares) and arctic Ivishak 2010 (blue triangles) populations. Bars indicate 95% CI

**FIGURE 5:**
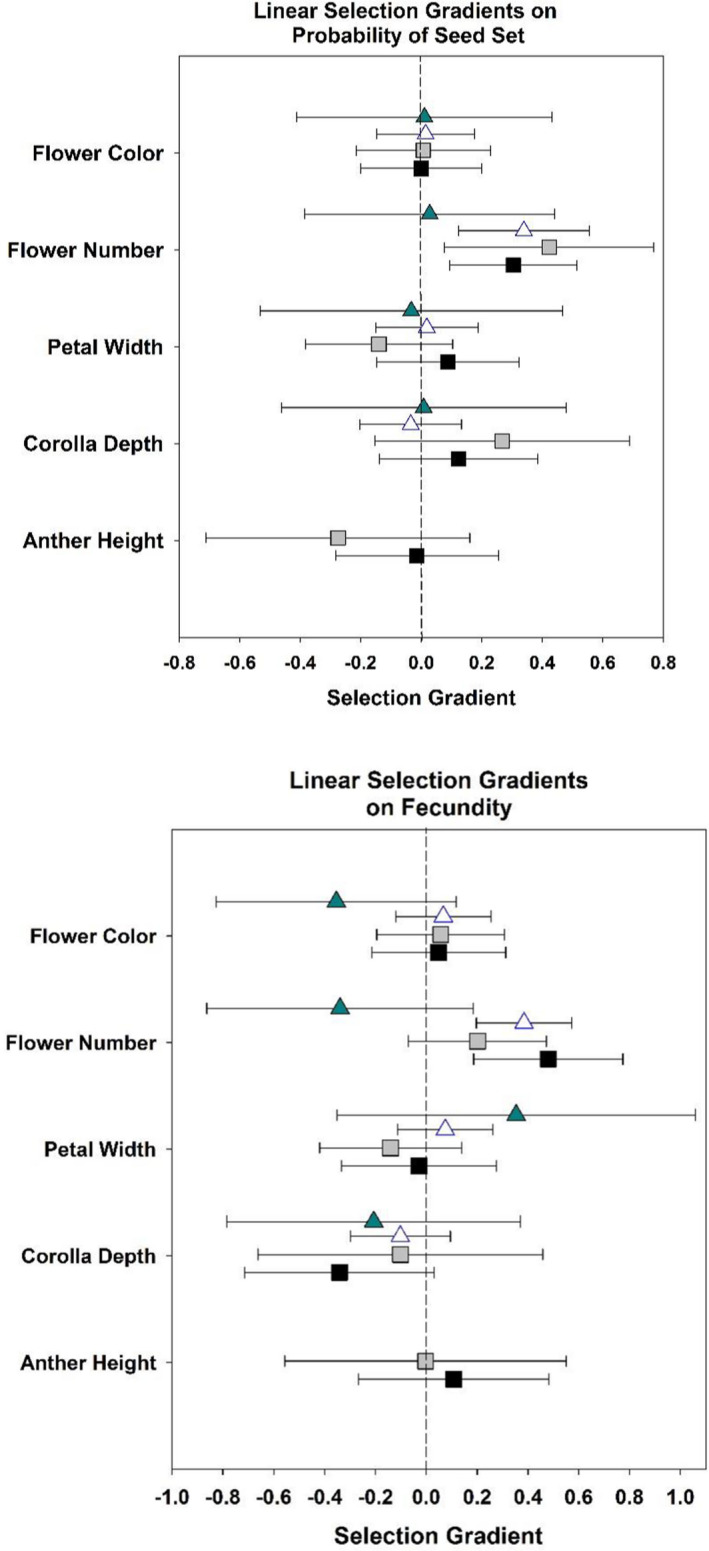
Variance‐standardized linear selection gradients (*β_σ_*) on probability of seed set (above) and fecundity (below) for all populations in 2009. Gradients for the arctic sites are Galbraith (solid turquoise triangle) and Ivishak (open blue triangle). Gradients for the subarctic sites are Eagle Summit (black squares) and Twelve‐Mile (gray squares). Anther height was not measured in the arctic populations. Bars indicate 95% CI

Selection gradients for all traits, populations, and open‐pollinated versus pollen‐augmented treatments are summarized in Figures 2, 3, 4, 5 and Tables 1A, 1B, 2, 3, 4. While we did not detect consistently stronger gradients across all traits in the pollinator‐mediated selection treatment relative to the pollen‐augmented treatment, we did observe a trend in stronger directional selection on increased flower number (Figure 2, Table 1A). Additionally, the interaction between flower color and anther height was under disruptive selection in open‐ but not pollen‐augmented plants (Figure 2b, Table 1B). Open‐pollinated plants with darker flowers and shorter anthers or plants with lighter flowers and more exserted anthers had higher probabilities of setting seed (Figure 3). Contrary to our prediction of pollinator‐mediated selection for increased pollinator attraction, we did not detect directional selection for larger petal size, greater pigmentation, or higher anther position in the open‐pollinated treatment (Figure 2, Table 1A).

**TABLE 1A:** Variance‐standardized linear (*β_σ_*) gradients (and 95% confidence intervals in parentheses) for open‐pollinated and pollen augmentation treatments using logistic regression on probability of seed set, and multiple linear regression on fecundity (seed number) for those individuals that did set seed at Eagle Summit in 2010

Trait	Probability of seed set		Fecundity	
	*β*_open_	*β*_augment_	*β*_open_	*β*_augment_
*L* (flower color)	0.00 (−0.20, 0.20)	0.04 (−0.06, 0.15)	0.05 (−0.22, 0.31)	−0.03 (−0.22, 0.15)
Flower number	0.31*** (0.09, 0.51)	0.13*** (0.02, 0.23)	0.48*** (0.18, 0.77)	0.29*** (0.11, 0.48)
Petal width	0.09 (−0.15, 0.32)	0.08 (−0.04, 0.20)	−0.03 (−0.34, 0.27)	−0.08 (−0.30, 0.14)
Corolla depth	0.12 (−0.14, 0.38)	−0.17*** (−**0.32,** −**0.02)**	−0.34* (−**0.72, 0.03)**	−0.01 (−0.30, 0.28)
Anther height	−0.01 (−0.28, 0.26)	0.04 (−0.07, 0.17)	0.10 (−0.27, 0.48)	0.06 (−0.23, 0.34)

Note

Gradients marginally and significantly different from 0 are shown in bold (*p < .10 > .05; **p < .05 > .01; ***p < .01). The regression model included only the five traits without interactions. Probability of seed set selection gradients is transformed from logistic regression coefficients using the method of Janzen and Stern (1998).

**TABLE 1B:** Variance‐standardized linear (*β_σ_*) and non‐linear (γ*_σ_*) selection gradients (and 95% confidence intervals in parentheses) for open‐pollinated and pollen augmentation treatments using logistic regression on probability of seed set, and multiple linear regression on fecundity (seed number) for those individuals that did set seed at Eagle Summit in 2010

Trait	Probability of seed set				Fecundity			
	*β*_open_	*β*_augmen_ *_t_*	*γ*_open_	*γ*_augment_	*β*_open_	*β*_augment_	*γ*_open_	*γ*_augment_
*L* (flower color)	−0.07 (−0.27, 0.13)	0.06 (−0.13, 0.26)	−0.08 (−0.46, 0.30)	0.28 (−0.10, 0.64)	0.01 (−0.41, 0.42)	−0.11 (−0.34, 0.13)	−0.22 (−0.57, 0.13)	−0.50 (−1.10, 0.10)
Flower number	0.33*** (0.12, 0.54)	0.18* **(−0.01, 0.38)**	−0.18 (−0.48, 0.14)	0.02 (−0.20, 0.22)	0.34 (−0.09, 0.77)	0.25** (0.04, 0.47)	0.34 (−0.32,1.00)	0.37* (−0.06, 0.80)
Petal width	0.12 (−0.10, 0.34)	−0.03 (−0.30, 0.25)	−0.004 (−0.40, 0.40)	0.26 (−0.12, 0.62)	−0.19 (−0.63, 0.24)	−0.12 (−0.40, 0.15)	0.15 (−0.22, 0.51)	−0.17 (−0.61, 0.27)
Corolla depth	0.17 (−0.12, 0.46)	−0.30*** (−0.57, −0.02)	−0.40 (−0.94, 0.16)	0.10 (−0.28, 0.50)	−0.15 (−0.89, 0.59)	−0.32 (−0.71, 0.08)	0.98 (−0.27, 2.23)	0.17 (−0.29, 0.63)
Anther height	−0.07 (−0.34, 0.21)	0.29*** (0.01, 0.56)	−0.36* (−0.74, 0.02)	−0.26* (−0.54, 0.02)	0.24 (−0.33, 0.82)	0.18 (−0.17, 0.52)	−0.31 (−0.88, 0.26)	1.16*** (0.77, 1.55)
*L* × Flower number			0.08 (−0.11, 0.28)	−0.18 (−0.42, 0.05)			−0.02 (−0.43, 0.39)	0.02 (−0.21, 0.26)
*L* × Petal width			−0.17 (−0.42, 0.08)	0.22 (−0.08, 0.51)			−0.05 (−0.48, 0.38)	−0.14 (−0.44, 0.16)
*L* × Corolla depth			−0.01 (−0.27, 0.25)	−0.05 (−0.31, 0.21)			−0.35 (−0.96, 0.27)	0.41** (0.02, 0.79)
*L* × Anther height			0.37*** (0.07, 0.67)	0.04 (−0.14, 0.22)			0.45 (−0.28, 1.18)	−0.19 (−0.56, 0.18)
Flower number × Petal width			0.04 (−0.25, 0.33)	−0.19 (−0.54, 0.15)			−0.14 (−0.82, 0.55)	−0.08 (−0.35, 0.19)
Flower number × Corolla depth			−0.11 (−0.42, 0.19)	−0.11 (−0.35, 0.13)			−0.74** (−1.42, −0.05)	−0.17 (−0.59, 0.25)
Flower number × Anther height			0.15 (−0.19, 0.49)	0.21 (−0.05, 0.47)			0.38 (−0.48, 1.24)	0.31* (−0.04, 0.66)
Petal width × Corolla depth			0.24 (−0.17, 0.65)	0.03 (−0.38, 0.44)			−0.21 (−1.10, 0.67)	0.58* (−0.09, 1.24)
Petal width × Anther height			−0.16 (−0.57, 0.25)	0.03 (−0.15, 0.21)			0.29 (−0.60, 1.17)	−0.45* (−0.96, 0.06)
Corolla depth × Anther height			0.22 (−0.13, 0.57)	−0.07 (−0.37, 0.23)			−0.24 (−0.33, 0.82)	−0.52 (−1.25, 0.21)

Note

Gradients marginally and significantly different from 0 are shown in bold (*p < .10 > .05; **p < .05 > 0.01; ***p < .01). Probability of seed set selection gradients is transformed from logistic regression coefficients using the method of Janzen and Stern (1998). Regression coefficients for *γ* matrix diagonals were multiplied by 2 to calculate concave and convex gradients. Positive *γ* values indicate concave (disruptive) selection, and negative values indicate convex (stabilizing) selection. The regression model included all five traits and fifteen cross‐product terms. Probability of seed set selection gradients is transformed from logistic regression coefficients using the method of Janzen and Stern (1998).

**TABLE 2:** Variance‐standardized linear (*β_σ_*) selection gradients (and 95% confidence intervals in parentheses) on probability of setting seed using logistic regression for open‐pollinated plants in arctic and subarctic regions

Trait	*β*_open_ Arctic			*β*_open_ Subarctic	
	Galbraith (2009) *n* = 40	Ivishak (2009) *n* = 64	Ivishak (2010) *n* = 57	Twelve‐Mile (2009) *n* = 42	Eagle Summit (2010) *n* = 129
*L* (flower color)	0.01 (−0.41, 0.43)	0.01 (−0.15, 0.18)	−0.03 (−0.16, 0.10)	0.01 (−0.21, 0.23)	0.00 (−0.20, 0.20)
Flower number	0.03 (−0.38, 0.44)	0.34*** (0.12, 0.56)	0.07 (−0.05, 0.18)	0.42** (0.08, 0.77)	0.31*** (0.09, 0.51)
Petal width	−0.03 (−0.53, 0.47)	0.02 (−0.15, 0.19)	−0.01 (−0.14, 0.12)	−0.14 (−0.38, 0.10)	0.09 (−0.15, 0.32)
Corolla depth	0.01 (−0.46, 0.49)	−0.03 (−0.20, 0.13)	0.18** (0.00, 0.35)	0.27 (−0.15, 0.69)	0.12 (−0.14, 0.38)
Anther height	**‐**	**‐**	0.10 (−0.06, 0.25)	−0.289 (−0.71, 0.16)	−0.01 (−0.3, 0.26)

Note

Gradients marginally and significantly different from 0 are shown in bold (*p < .10 > .05; **p < .05 > .01; ***p < .01). Anther height was not recorded for Galbraith and Ivishak populations in 2009. Selection gradients are transformed from logistic regression coefficients using the method of Janzen and Stern (1998).

**TABLE 3:** Variance‐standardized linear (*β_σ_*) selection gradients (and 95% confidence intervals in parentheses) on fecundity for open‐pollinated plants in arctic and subarctic regions

Trait	*β*_open_ Arctic			*β*_open_ Subarctic	
	Galbraith (2009) *n* = 14	Ivishak (2009) *n* = 42	Ivishak (2010) *n* = 50	Twelve‐Mile (2009) *n* = 26	Eagle Summit (2010) *n* = 55
*L* (flower color)	−0.37 (−0.84, 0.10)	0.07 (−0.12, 0.26)	−0.27** (−0.49, −0.05)	0.05 (−0.20, 0.31)	0.04 (−0.22, 0.31)
Flower number	−0.35 (−0.88, 0.17)	0.39*** (0.20, 0.58)	0.30*** (0.09, 0.53)	0.20 (−0.07, 0.47)	0.48*** (0.18, 0.77)
Petal width	0.34 (−0.36, 1.05)	0.08 (−0.11, 0.27)	−0.10 (−0.27, 0.071)	−0.14 (−0.42, 0.14)	−0.03 (−0.34, 0.27)
Corolla depth	−0.22 (−0.80, 0.36)	−0.10 (−0.30, 0.10)	−0.10 (−0.36, 0.17)	−0.10 (−0.66, 0.92)	−0.34* (−0.72, 0.03)
Anther height	**‐**	**‐**	0.10 (−0.24, 0.34)	0.00 (−0.55, 0.55)	0.10 (−0.27, 0.48)

Note

Gradients marginally and significantly different from 0 are shown in bold (*p < .10 > .05; **p < .05 > .01; ***p < .01). Anther height was not recorded for Galbraith and Ivishak populations in 2009. Selection gradients are transformed from logistic regression coefficients using the method of Janzen and Stern (1998).

**TABLE 4:** Variance‐standardized linear (*β*_open_) and non‐linear (γ_open_) selection gradients (and 95% confidence intervals in parentheses) for open‐pollinated plants using logistic regression on probability of seed set, and multiple linear regression on fecundity (seed number) for those individuals that did set seed at Ivishak in 2010

Traits	Probability of Seed Set	Fecundity	
	*β*_open_	*β*_open_	*γ*_open_
*L* (flower color)	−0.027 (−0.16, 0.10)	**−0.25·(−0.50, 0.01)**	0.02 (−0.61, 0.66)
Flower number	0.07 (−0.05, 0.18)	0.56** (0.33, 0.80)	0.06 (−0.34, 046)
Petal width	−0.01 (−0.14, 0.12)	0.07 (−0.20, 0.32)	0.10 (−0.34, 0.24)
Corolla depth	0.18* (0.002, 0.35)	−0.15 (−0.49, 0.19)	0.29 (−0.37, 0.95)
Anther height	0.10 (−0.06, 0.25)	0.30 (−0.06, 0.66)	0.16 (−0.07, 0.40)
*L* × Flower number			**−0.25·(−0.54, 0.04)**
*L* × Petal width			0.05 (−0.26, 0.33)
*L* × Corolla depth			0.30 (−0.07, 0.67)
*L* × Anther height			−0.30 (−0.66, 0.08)
Flower number × Petal width			0.16 (−0.19, 0.52)
Flower number × Corolla depth			0.11 (−0.26, 0.48)
Flower number × Anther height			0.09 (−0.29, 0.48)
Petal width × Corolla depth			−0.05 (−0.50, 0.40)
Petal width × Anther height			0.00 (−0.43, 0.44)
Corolla depth × Anther height			−0.12 (−0.58, 0.34)

Note

Gradients marginally and significantly different from 0 are shown in bold (·*p* < .10 > .05; *p < .05 > .01; **p < .01). Regression coefficients for *γ* matrix diagonals were multiplied by 2 to calculate concave and convex gradients. The regression model for fitness estimated by probability of seed set included only the five traits, as most individuals set seed in this year and site, limiting confidence in estimates of regression coefficients. The regression model of fitness estimated through fecundity, however, had sufficient sample size to include all five traits and fifteen cross‐product terms. Probability of seed set selection gradients is transformed from logistic regression coefficients using the method of Janzen and Stern (1998).

In the pollen‐augmented treatment, the probability of seed set was greater for individuals with shorter corolla tubes (Figure 2, Table 1A). For those individuals that set seed in the pollen augmentation treatment, fecundity was also lowest for individuals with intermediate anther position (i.e., disruptive selection, Figure 1, Table 1B).

Our second prediction was that phenotypic selection gradients for open‐pollinated plants would be of greater magnitude in arctic populations relative to subarctic populations. We find little support for this hypothesis, with strong directional selection observed for some traits in arctic sites and strong directional selection for other traits in subarctic sites in both 2009 and 2010 (Figures 4 and 5; Tables 2 and 3). Flower number was under significant positive linear selection for the majority of populations (Figure 5; Table 3). Selection gradients in 2010 open pollination treatments at the arctic Ivishak and subarctic Eagle Summit populations indicate consistent directional selection on greater flower number, and an indication for potential directional selection for shorter corolla tubes, and higher anther position (Figure 4). When measuring fitness as the probability of seed set, however, selection was not detectable in 2010 when 85% of flowering individuals at Ivishak set seed (Table 2). Strong directional selection was observed for increased flower number at Ivishak in 2010 when measuring fitness in terms of fecundity, however (Figure 4, Table 3). Directional selection for darker flowers was observed for the arctic population (Ivishak), but not the subarctic population (Eagle Summit) in 2010 (Figure 4, Table 3). The arctic population displayed concave (disruptive) selection for the interaction of “flower color” and “corolla depth,” with individuals of intermediate combinations of trait values displaying reduced fecundity (Table 4).

## 4 DISCUSSION

### 4.1 Natural selection under arctic and subarctic environments

Selection on floral traits is predicted to be strongest under pollen‐limited environments where traits that increase pollinator attractiveness or improve selfing are expected to be favored (see Ashman & Morgan, 2004; Kalisz et al., 2004; Harder & Aizen, 2010; Teixido & Aizen, 2019). Phenotypic selection for attractive floral traits in pollen‐limited environments has been found in some systems (Caruso, 2000; Johnston, 1991; Totland, 2001; Trunschke et al., 2017), but not in others (see Teixido & Aizen, 2019; Totland, 2004; Souto‐Vilarós et al., 2018), and is understood to depend on the ecological context and species characteristics (Harder & Aizen, 2010). In a New Zealand alpine plant, the strength of selection on flower color (whiter flowers had greater fitness) was stronger under a lower pollination limitation treatment than when pollen was more limiting (Campbell & Bischoff, 2013); however, in this case non‐pollinator‐mediated selection was invoked. Different populations were also demonstrated to vary in magnitude of selection on flower size, with positive directional selection in populations with lower reproductive assurance in a Mediterranean plant (Teixido & Aizen, 2019).

Selection on floral traits can also occur when pollen limitation is absent (Galen, 1996; Parachnowitsch & Kessler, 2010). In this study of *P*. *nudicaulis*, while we did not detect consistently stronger gradients across all traits in the pollinator‐mediated selection treatment, selection gradients were generally stronger under the natural environment relative to the pollen augmentation treatment, where pollinator‐mediated selection should be largely removed. Other sources of selection would of course continue to occur.

Selection gradients at the arctic Ivishak population were also stronger in the year with less favorable weather and much lower natural seed set. The 2009 flowering season at Ivishak was marked with a wet, windy, and cold climate that would likely limit insect flight time and pollinator availability (Bergman et al., 1996; Totland, 1994). In contrast, the 2010 season was sunny with warmer temperatures and *ad lib* observations suggested increased pollinator activity. While we did not specifically test for pollen limitation at this population, seed set in 2010 was comparable to hand pollination treatments in the subarctic alpine sites to the south.

Strong positive linear selection for a greater number of flowers was found for nearly all open‐pollinated populations, as well as the pollen augmentation treatment. Greater flower number may be influencing the probability of seed set by increased opportunities for pollen receipt through a reproductive season that typically has many days with unfavorable weather, as well as through attracting a greater number of pollinators to a larger and more rewarding floral display. Additionally, our selection results may be underestimates since they do not include the male component of fitness. Male fitness is also expected to increase with increasing number of flowers; unfortunately, selection on male fitness is rarely studied, despite its importance (Sutherland & Delph, 1984). Plants containing a greater number of ovules than are on average fertilized have been hypothesized to benefit from occasional “jackpot” chance visits in environments with highly stochastic pollinator visits (Ashman et al., 2004; Burd et al., 2009). Pollinator visits to *P*. *nudicaulis* in tundra habitats occur at much lower rates and depend on windows of favorable climate compared to plants in temperate habitats (Fulkerson et al., 2012). Indeed, an increase in ovule number would be beneficial for occasional pollinator visits, but an increase in flower number would further enhance the probability of seed set for an individual through geitonogamy, as well as presumably promoting pollen export (male fitness).

Selection gradients based on probability of seed set and fecundity appeared to be of greater magnitude for flower number in open‐pollinated plants than in pollen‐augmented plants, suggesting pollinators were either discriminating between inflorescence sizes or if larger inflorescences have receptive flowers for a longer time period, they are more likely to be visited. In this arctic and subarctic context, both attraction of insect pollinators that are not abundant and diverse (Ollerton, 2017), and extending the flowering period may be particularly important. Larger inflorescences, however, may also increase herbivory and seed predation (Caruso et al., 2019; Galen, 1999). Overall, phenotypic selection for a greater number of flowers in plants appears to be common in other floral selection studies and is expected as the trait is directly tethered to fitness metrics (reviewed in Caruso et al., 2019; Harder & Johnson, 2009; Parachnowitsch & Kessler, 2010).

The greater strength of pollinator‐mediated, relative to non‐pollinator‐mediated, selection on flower number is consistent with our prediction of selection favoring traits associated with enhanced pollinator attraction. Contrary to our prediction, however, we did not detect selection for larger petal size. Pollinators have been shown to prefer flowers with larger petals and corollas in a number of other studies (Ashman et al., 2004; Campbell et al., 1996; Galen, 1996; Gómez, 2003; Parachnowitsch & Kessler, 2010; Sandring & Ågren, 2009; Sletvold & Ågren, 2010). However, the pollinator guilds of the arctic and subarctic habitats are moderately diverse, generally dominated by flies, and dissimilar from previously studied regions (Fulkerson et al., 2012; Tiusanen et al., 2016; Tiusanen et al., 2019), and phenotypic selection is typically higher in plants pollinated by bees, long‐tongued flies, or birds (Caruso et al., 2019). Additionally, directional selection on the size of the corolla or pollination unit (e.g., capitulum in Asteraceae) has not always been detected (Parachnowitsch et al., 2012), even when seed set is significantly pollen limited (Andersson & Widén, 1992; Sletvold et al., 2010). It is possible that some of the apparent phenotypic selection on flower size observed in these studies could be a product of covariation in ovule number (see Hansen et al., 2003; however, see Stanton & Preston, 1988). In *P*. *nudicaulis*, we suspect that flower size has a minor impact on the overall floral display perceived by pollinators and unmeasured traits such as scent production may be significantly more important in pollinator perception (Parachnowitsch et al., 2012).

An alternative prediction to selection for enhanced pollinator attraction in pollen‐limited environments could be selection for increased capacity for selfing. *Parrya nudicaulis* is protandrous and largely pollinator dependent, but a small frequency of flowers that had pollinators excluded do set seed (Fulkerson et al., 2012); thus, it suggests that sufficient phenotypic variation exists in traits associated with selfing to respond to selection in these populations. We did not, however, detect directional selection on reduced petal size, lower anther position, or reduced floral pigmentation in open‐pollinated plants in either arctic or subarctic populations. It is also possible for herbivores, seed predators, and resource costs to generate selection on reduced floral displays (Caruso et al., 2019; Descamps et al., 2021; Galen, 1999). The arctic population experienced directional selection for more darkly pigmented flowers; although as we did not pair a pollen augmentation treatment with the open‐pollinated plants at this population, we are not able to attribute the agent of selection to pollinators or another source. Anther–stigma separation was not measured to avoid accidental hand pollination or damage to the stigma, but we measured corolla tube length which is correlated with stigma position (Fulkerson et al., 2012) and anther height. Our results suggest modest directional selection for shorter corolla tube length at both the arctic and subarctic populations and for higher anther position at the arctic Ivishak site. Shorter corolla tubes are expected to be associated with a lower stigma position (below the top four anthers at anthesis) and more likely to receive self‐pollen; however, it is not clear that shorter tube length is indeed associated with greater reproductive assurance in this species. Selection on timing of male and female receptivity may be more important than variation in proximity of anthers and stigmas, as well as corolla size, on the capacity for self‐fertilization in the absence of pollinators since this species is protandrous (Fulkerson et al., 2012).

We observed selection acting on combinations of floral traits in the open pollination treatment that were not observed in the pollen augmentation treatment. Notably, fitness was greater in plants with darker flowers and less exserted anthers or in plants with lighter flowers and more exserted in the open‐pollinated treatment. The cause of the interaction in trait values is unknown, but could include divergent selective pressures imposed by different pollinator guilds. In general, high within‐population variation in floral traits could be maintained by divergent or fluctuating selection on combinations of partially unlinked traits, as may be the case for flower color and anther position in *P*. *nudicaulis*.

We predicted that the magnitude of phenotypic selection would be greater on floral traits in arctic populations relative to subarctic populations. The strength of selection should increase with increasing pollen limitation (Caruso et al., 2019; Trunschke et al., 2017) and pollen limitation is believed to increase at higher latitudes (and altitudes) as weather and climate appropriate for pollinator service declines (Bergman et al., 1996; Inouye, 2020; Totland, 1994). In a number of cases we detected greater selection in the arctic populations; however, the strength of selection was inconsistent among populations, traits, and years. Often the direction of linear selection was divergent for traits (or trait combinations) between the arctic and subarctic populations. The arctic populations are approximately 400 km to the north of the subarctic populations and have substantially lower mean July temperatures on average (Dick et al., 2011) that would be expected to be associated with reduced pollinator activity; however, the subarctic sites are at a higher elevation and are also often subjected to inclement weather. Year‐to‐year variation in weather is likely to make detection of regional patterns in selection gradients difficult to detect.

We provide modest evidence of stronger selection gradients for pigmentation at the higher latitudes compared to the lower‐latitude sites, where darker violet individuals had higher fecundity. Indeed, at a population level, anthocyanin pigmentation of *P*. *nudicaulis* increases in frequency with increasing latitude (Dick et al., 2011). Flower color did not affect the probability of seed set at the Ivishak population, but selection coefficients for darker flower color were strongly significant with a greater number of flowers and marginally on its own. Flower color did not enhance the probability of seed set unless it interacted with another trait in the other sites. Selection on flower color can not only be a result of herbivores, pathogens, or abiotic factors directly acting on the trait but also be a result of indirect selection through correlated traits (Campbell & Bischoff, 2013; Frey, 2004; Rausher, 2008; Strauss & Whittall, 2006). Selection on flower color appeared to be operating through interactions with other floral characters, suggesting flower color is being indirectly selected by pollinators through correlated traits or other unmeasured traits or directly selected by abiotic responses not measured in this experiment.

Greater anthocyanin concentrations in higher latitudes and elevations would likely enhance growth and survivorship from the abiotic stresses associated with these habitats. Anthocyanins are important components for osmotic regulation in drought and frost‐like conditions and protect plant cells from visible light by screening it through attenuation (Close & Beadle, 2003). A combination of many abiotic selective pressures interacting with genetic adaptations may be responsible for color variation between higher‐ and lower‐latitude populations of *P*. *nudicaulis*. We envision future studies on phenotypic selective pressures to incorporate several years of measurements since reproductive success varied greatly between the years for the Ivishak site, suggesting either resource limitation or pollinator reduction resulting in pollen limitation.

## AUTHOR CONTRIBUTIONS

**Matthew L. Carlson:** Conceptualization (lead); data curation (supporting); formal analysis (equal); funding acquisition (lead); investigation (equal); methodology (equal); project administration (equal); resources (equal); software (equal); supervision (lead); validation (equal); visualization (lead); writing – original draft (equal); writing – review and editing (lead). **Justin R. Fulkerson:** Conceptualization (supporting); data curation (lead); formal analysis (equal); funding acquisition (supporting); investigation (equal); methodology (equal); project administration (equal); resources (equal); software (equal); supervision (supporting); validation (equal); visualization (supporting); writing – original draft (equal); writing – review and editing (supporting).

## Supporting information

Table S1‐S5

## Data Availability

Data will be accessible to the public through our Alaska Center for Conservation Science, University of Alaska data portal (https://accs.uaa.alaska.edu/ and https://accscatalog.uaa.alaska.edu/search/type/dataset), where we serve diverse sets of biological and conservation data for Alaska and the Arctic. Voucher specimens are deposited at the UAAH herbarium and are available digitally at https://www.pnwherbaria.org/data/search.php.
