# Supplementary material for: Phenotypic selection on floral traits in the arctic plant Parrya nudicaulis (Brassicaceae)
Source: Ecol Evol. 2022 Mar 1;12(3):e8624. doi: 10.1002/ece3.8624 (PMC8888260; doi:10.1002/ece3.8624)
Supplement: Supplementary file 1 — Table S1‐S5 [file ECE3-12-e8624-s001.docx]

**Supplemental Table 1.** Populations in arctic and subarctic regions in which phenotypic selection was measured for open-pollinated plants and pollen augmented plants. Sample size is indicated by “*n*”; “n/a” indicates that no phenotypic selection estimation was made for that population in the particular year).

| **Year** | **Arctic Populations** | |  | **Subarctic Populations** | |
| --- | --- | --- | --- | --- | --- |
|  | **Ivishak**  (69° 20' N, 148° 45' W, 280 m elev.) | **Galbraith**  (68°27' N, 149°33' W, 880 m elev.) |  | **Eagle Summit**  (65° 28’N, 145° 25’W, 1100 m elev.) | **Twelve Mile Summit**  (65° 24' N, 145° 44' W, 680 m elev.) |
| **2009** | Open Pollination  *n* = 64 | Open Pollination  *n* = 41 |  | n/a | Open Pollination  *n* = 42 |
| **2010** | Open Pollination  *n* = 57 | n/a |  | Open Pollination  *n* = 129 | n/a |
|  |  |  |  | Pollen Augmentation  *n* = 83 |  |

**Supplemental Table 2.** Descriptive statistics of floral traits measured for estimates of phenotypic selection in subarctic and arctic populations. Values with “n/a” indicate traits were not measured and not included in selection analysis, “*n*” indicates sample size.

| **Site** | **Year** | **Statistic** | **L* (Flower Color)** | **Flower Number** | **Petal Width (mm)** | **Corolla Depth (mm)** | **Anther Height (mm)** |
| --- | --- | --- | --- | --- | --- | --- | --- |
| Ivishak | 2009 | *n* | 64 | 64 | 64 | 63 | n/a |
|  |  | Mean | 81.4 | 9.9 | 9.16 | 7.72 | n/a |
|  |  | Standard Error | 1.17 | 0.37 | 0.22 | 0.11 | n/a |
|  |  | Standard Deviation | 9.36 | 3.01 | 1.73 | 0.9 | n/a |
|  |  | Sample Variance | 87.79 | 9.1 | 3.01 | 0.82 | n/a |
| Galbraith | 2009 | *n* | 40 | 40 | 40 | 40 | n/a |
|  |  | Mean | 80.6 | 9.65 | 8.07 | 7.44 | n/a |
|  |  | Standard Error | 1.45 | 0.31 | 0.22 | 0.15 | n/a |
|  |  | Standard Deviation | 9.23 | 1.99 | 1.38 | 0.977 | n/a |
|  |  | Sample Variance | 85.22 | 3.98 | 1.9 | 0.95 | n/a |
| 12Mile | 2009 | *n* | 55 | 56 | 56 | 56 | n/a |
|  |  | Mean | 86.58 | 12.98 | 9.34 | 10.95 | n/a |
|  |  | Standard Error | 1.64 | 0.39 | 0.22 | 0.15 | n/a |
|  |  | Standard Deviation | 12.17 | 2.92 | 1.69 | 1.6 | n/a |
|  |  | Sample Variance | 148.3 | 8.52 | 2.86 | 1.35 | n/a |
| Ivishak | 2010 | *n* | 58 | 58 | 57 | 57 | 57 |
|  |  | Mean | 86.69 | 10 | 9.04 | 8.76 | 9.32 |
|  |  | Standard Error | 1.37 | 0.38 | 0.2 | 0.12 | 0.11 |
|  |  | Standard Deviation | 10.44 | 2.92 | 1.52 | 0.92 | 0.82 |
|  |  | Sample Variance | 109.09 | 8.52 | 2.3 | 0.84 | 0.67 |
| 12Mile | 2010 | *n* | 42 | 42 | 42 | 41 | 40 |
|  |  | Mean | 86.81 | 9.19 | 9.38 | 9.39 | 9 |
|  |  | Standard Error | 1.99 | 0.55 | 0.23 | 0.13 | 0.13 |
|  |  | Standard Deviation | 12.94 | 3.58 | 1.49 | 0.83 | 0.85 |
|  |  | Sample Variance | 167.57 | 12.84 | 2.23 | 0.69 | 0.73 |
| Eagle Summit (Open Pollination) | 2010 | *n* | 128 | 124 | 128 | 128 | 127 |
|  |  | Mean | 88.09 | 7.79 | 9.39 | 9.41 | 8.33 |
|  |  | Standard Error | 1.07 | 0.27 | 0.13 | 0.08 | 0.09 |
|  |  | Standard Deviation | 12.11 | 3.06 | 1.42 | 0.98 | 1.01 |
|  |  | Sample Variance | 146.78 | 9.37 | 2.03 | 0.96 | 1.01 |
| Eagle Summit (Pollen Augmentation) | 2010 | *n* | 84 | 84 | 84 | 84 | 84 |
|  |  | Mean | 87 | 7.82 | 9.23 | 9.41 | 8.31 |
|  |  | Standard Error | 1.28 | 0.31 | 0.13 | 0.1 | 0.09 |
|  |  | Standard Deviation | 11.78 | 2.8 | 1.24 | 0.98 | 0.9 |
|  |  | Sample Variance | 138.82 | 8.19 | 1.54 | 0.97 | 0.81 |

**Supplemental Table 3A.** Mean-standardized linear (*β_μ_*) gradients (and 95% confidence intervals in parentheses) for open-pollinated and pollen augmentation treatments using logistic regression on probability of seed set, and multiple linear regression on fecundity (seed number) for those individuals that did set seed at Eagle Summit in 2010. Gradients marginally and significantly different from zero are shown in bold (**·** = *p* < 0.10 > 0.05; ***** = *p* < 0.05 > 0.01; ****** = *p* < 0.01). The regression model included only the five traits without interactions. Probability of seed set selection gradients are transformed from logistic regression coefficients using the method of Janzen & Stern (1998).

|  | *Probability of Seed Set* | |  | *Fecundity* | |
| --- | --- | --- | --- | --- | --- |
| Trait | *β_open_* | *β_augment_* |  | *β_open_* | *β_augment_* |
| L (Flower Color) | 0.00  (-1.42, 1.42) | 0.31  (-0.46, 1.08) |  | 0.33  (-1.51, 2.18) | -0.26  (-1.64, 1.13) |
|  |  |  |  |  |  |
| Flower Number | **0.78****  **(0.24, 1.31)** | **0.37****  **(0.07, 0.67)** |  | **1.65****  **(0.64, 2.67)** | **0.84****  **(0.31, 1.37)** |
|  |  |  |  |  |  |
| Petal Width | 0.58  (-0.97, 2.13) | 0.60  (-0.29, 1.49) |  | -0.21  (-2.31, 1.89) | -0.63  (-2.26, 1.00) |
|  |  |  |  |  |  |
| Corolla Depth | 1.18  (-1.33, 3.70) | **-1.16****  **(-3.04, -0.22)** |  | **-3.28·**  **(-6.83, 0.27)** | -0.09  (-2.84, 2.66) |
|  |  |  |  |  |  |
| Anther Height | -0.11  (-2.348, 2.11) | 0.41  (-0.73, 1.54) |  | 0.93  (-2.34, 4.19) | 0.52  (-2.07, 3.09) |
|  |  |  |  |  |  |

**Supplemental Table 3B.** Mean-standardized linear (*β_μ_*) and nonlinear (γ*_μ_*) selection gradients (and 95% confidence intervals in parentheses) for open-pollinated and pollen augmentation treatments using logistic regression on probability of seed set, and multiple linear regression on fecundity (seed number) for those individuals that did set seed at Eagle Summit in 2010. Gradients marginally and significantly different from zero are shown in bold (· = *p* < 0.10 > 0.05; ***** = *p* < 0.05 > 0.01; ****** = *p* < 0.01). Probability of seed set selection gradients are transformed from logistic regression coefficients using the method of Janzen & Stern (1998). Regression coefficients for *γ* matrix diagonals were multiplied by 2 to calculate concave and convex gradients. The regression model included all five traits and fifteen cross-product terms. Probability of seed set selection gradients are transformed from logistic regression coefficients using the method of Janzen & Stern (1998).

|  | *Probability of Seed Set* | | | | |  | *Fecundity* | | | | |
| --- | --- | --- | --- | --- | --- | --- | --- | --- | --- | --- | --- |
| Trait | *β_open_* | *β_augment_* |  | *γ_open_* | *γ_augment_* |  | *β_open_* | *β_augment_* |  | *γ_open_* | *γ_augment_* |
| L (Flower Color) | -0.50  (-1.94, 0.94) | 0.45  (-0.98, 1.88) |  | -4.1  (-23.3, 15.1) | 15.1  (-5.1, 35.2) |  | 0.07  (-2.9, 3.0) | -0.81  (-2.50, 0.95) |  | -21.7  (-56.2, 12.8) | -54.1  (-119, 10.8) |
|  |  |  |  |  |  |  |  |  |  |  |  |
| Flower Number | **0.85****  **(0.31, 1.38)** | **0.52**·  **(-0.03, 1.38)** |  | -1.13  (-3.2, 0.9) | 0.14  (-1.54, 1.83) |  | 0.98  (-0.26, 2.22) | **0.71***  **(0.11, 1.33)** |  | 5.66  (0.17,11.3) | **5.99·**  **(-0.97, 12.9)** |
|  |  |  |  |  |  |  |  |  |  |  |  |
| Petal Width | 0.80  (-0.65, 2.26) | -0.21  (-2.23, 1.82) |  | -0.20  (-17.6, 17.2) | 14.0  (-6.3, 34.3) |  | -1.31  (-4.33, 1.65) | -0.89  (-2.96, 1.11) |  | 14.2  (-20.8, 48.1) | -18.6  (-66.9, 87.7) |
|  |  |  |  |  |  |  |  |  |  |  |  |
| Corolla Depth | 1.62  (-1.18, 4.42) | **-2.79****  **(-5.43, -0.15)** |  | -36.6  (-87.8, 14.4) | 9.6  (-26.2, 45.5) |  | -1.43  (-8.50, 5.64) | -3.05  (-6.76, 0.76) |  | 179.0  (-63.9, 294) | 30.8  (-52.6, 114) |
|  |  |  |  |  |  |  |  |  |  |  |  |
| Anther Height | -0.55  (-2.83, 1.73) | **2.63****  **(0.10, 5.17)** |  | **-24.1·**  **(-50.8, 2.5)** | **-21.3·**  **(-44.4, 1.8)** |  | 2.10  (-2.88,7.15) | 1.64  (-1.56, 4.76) |  | -47.2  (-134, 39.5) | **194****  **(129, 260)** |
|  |  |  |  |  |  |  |  |  |  |  |  |
| L × Flower Number |  |  |  | 3.0  (-4.1, 10.2) | -7.7  (-17.5, 2.1) |  |  |  |  | -0.81  (-17.4, 15.8) | 0.84  (-8.79, 10.9) |
|  |  |  |  |  |  |  |  |  |  |  |  |
| L × Petal Width |  |  |  | -16.2  (-40.0, 7.6) | 23.4  (-9.0, 55.8) |  |  |  |  | -4.82  (-46.3, 36.6) | -15.3  (-47.9, 17.4) |
|  |  |  |  |  |  |  |  |  |  |  |  |
| L × Corolla Depth |  |  |  | -1.1  (-37.1, 34.9) | -6.4  (-42.8, 30.0) |  |  |  |  | -46.9  (-128, 36.2) | **57.5***  **(2.8, 111)** |
|  |  |  |  |  |  |  |  |  |  |  |  |
| L × Anther Height |  |  |  | **43.8****  **(9.0, 78.6)** | 5.6  (-18.4, 29.7) |  |  |  |  | 55.1  (-34.3, 144) | -25.6  (-75.4, 24.2) |
|  |  |  |  |  |  |  |  |  |  |  |  |
| Flower Number ×  Petal Width |  |  |  | 1.33  (-8.6, 11.2) | -8.2  (-22.8, 6.4) |  |  |  |  | -5.55  (-32.5, 21.8) | -3.37  (-14.7, 8.0) |
|  |  |  |  |  |  |  |  |  |  |  |  |
| Flower Number ×  Corolla Depth |  |  |  | -5.61  (-20.5, 9.3) | -6.1  (-19.0, 6.9) |  |  |  |  | **-40.8***  **(-78.3, -2.76)** | -9.21  (-32.0, 13.6) |
|  |  |  |  |  |  |  |  |  |  |  |  |
| Flower Number ×  Anther Height |  |  |  | 6.46  (-7.9, 20.8) | 10.8  (-2.7, 24.2) |  |  |  |  | 19.1  (-24.1, 62.4) | **16.1·**  **(-2.1, 34.4)** |
|  |  |  |  |  |  |  |  |  |  |  |  |
| Petal Width ×  Corolla Depth |  |  |  | 30.7  (-20.1, 82.3) | 4.2  (-53.5, 62.0) |  |  |  |  | -27.6  (-144, 87.9) | **81.8·**  **(-12.7, 175)** |
|  |  |  |  |  |  |  |  |  |  |  |  |
| Petal Width ×  Anther Height |  |  |  | -17.3  (-61.8, 27.1) | 4.2  (-20.4, 28.8) |  |  |  |  | 34.7  (-71.8, 140) | **-61.0·**  **(-130, 8.13)** |
|  |  |  |  |  |  |  |  |  |  |  |  |
| Corolla Depth ×  Anther Height |  |  |  | 34.7  (-20.7, 90.1) | -12.5  (-64.8, 39.9) |  |  |  |  | -40.0  (-165, 83.3) | -90.7  (-217, 36.6) |

**Supplemental Table 4.** Mean-standardized linear (*β_μ_*) selection gradients (and 95% confidence intervals in parentheses) on probability of setting seed using logisitic regression for open-pollinated plants in arctic and subarctic regions. Gradients marginally and significantly different from zero are shown in bold (· = *p* < 0.10 > 0.05; ***** = *p* < 0.05 > 0.01; ****** = *p* < 0.01). Anther height was not recorded for Galbraith and Ivishak populations in 2009. Selection gradients are transformed from logistic regression coefficients using the method of Janzen & Stern (1998).

|  | *β_open_* Arctic | | | | | |  | *β_open_* Subarctic | | | |
| --- | --- | --- | --- | --- | --- | --- | --- | --- | --- | --- | --- |
| Trait | Galbraith  (2009)  *n* = 40 | | Ivishak  (2009)  *n* = 64 | | Ivishak  (2010)  *n* = 57 | |  | 12 Mile  (2009)  *n* = 42 | | Eagle  (2010)  *n* = 129 | |
| L (Flower Color) | 0.09  (-3.60, 3.79) | 0.13  (-1.29, 1.54) | | -0.24  (-1.36, 0.89) | |  | 0.04  (-1.44, 1.53) | | 0.00  (-1.42, 1.42) | |  |
| Flower Number | 0.13  (-1.86, 2.13) | **1.11****  **(0.40, 1.83)** | | 0.23  (-0.15, 0.63) | |  | **1.32***  **(0.24, 2.40)** | | **0.78****  **(0.24, 1.31)** | |  |
| Petal Width | -0.19  (-3.11, 2.73) | 0.10  (-0.79, 1.00) | | -0.04  (-0.80, 0.72) | |  | -0.88  (-2.41, 0.65) | | 0.58  (-0.97, 2.13) | |  |
| Corolla Depth | 0.06  (-3.52, 3.65) | -0.30  (-1.74, 1.14) | | **1.69***  **(0.02, 3.35)** | |  | 3.01  (-1.72, 7.75) | | 1.18  (-1.33, 3.70) | |  |
| Anther Height | **-** | **-** | | 1.11  (-0.63, 2.85) | |  | -2.89  (-7.47, 1.68) | | -0.11  (-2.348, 2.11) | |  |

**Supplemental Table 5.** Mean-standardized linear (*β_μ_*) selection gradients (and 95% confidence intervals in parentheses) on fecundity for open-pollinated plants in arctic and subarctic regions. Gradients marginally and significantly different from zero are shown in bold (· = *p* < 0.10 > 0.05; ***** = *p* < 0.05 > 0.01; ****** = *p* < 0.01). Anther height was not recorded for Galbraith and Ivishak populations in 2009. Selection gradients are transformed from logistic regression coefficients using the method of Janzen & Stern (1998).

|  | *β_open_* Arctic | | | | | |  | *β_open_* Subarctic | | | |
| --- | --- | --- | --- | --- | --- | --- | --- | --- | --- | --- | --- |
| Trait | Galbraith  (2009)  *n* = 14 | | Ivishak  (2009)  *n* = 42 | | Ivishak  (2010)  *n* = 50 | |  | 12 Mile  (2009)  *n* = 26 | | Eagle  (2010)  *n* = 55 | |
| L (Flower Color) | -2.77  (-6.12, 0.78) | 0.60  (-1.01, 2.21) | | **-2.34***  **(-4.25, -0.41)** | |  | 0.47  (-1.70, 2.64) | | 0.33  (-1.51, 2.18) | |  |
| Flower Number | -1.74  (-4.33, 0.84) | **1.43****  **(0.74 2.12)** | | **1.01****  **(0.30, 1.83)** | |  | 0.69  (-0.25, 1.64) | | **1.65****  **(0.64, 2.67)** | |  |
| Petal Width | 2.02  (-2.19, 6.24) | 0.42  (-0.58, 1.41) | | -0.61  (-1.63, 0.43) | |  | -0.85  (-2.52, 0.82) | | -0.21  (-2.31, 1.89) | |  |
| Corolla Depth | -1.59  (-5.73, 2.55) | -0.82  (-2.45, 0.81) | | -1.00  (-3.69, 1.68) | |  | -1.05  (-6.73, 4.64) | | **-3.28·**  **(-6.83, 0.27)** | |  |
| Anther Height | **-** | **-** | | 1.06  (-2.46, 3.43) | |  | -0.04  (-5.66, 5.57) | | 0.93  (-2.34, 4.19) | |  |
